# Incidence of back pain in adolescent athletes: a prospective study

**DOI:** 10.1186/s13102-016-0064-7

**Published:** 2016-12-07

**Authors:** Steffen Mueller, Juliane Mueller, Josefine Stoll, Olaf Prieske, Michael Cassel, Frank Mayer

**Affiliations:** 1 University Outpatient Clinic, Sports Medicine & Sports Orthopaedics, University of Potsdam, Am Neuen Palais 10 - House 12, D-14469 Potsdam, Germany; 2 Division of Training and Movement Sciences, University of Potsdam, Potsdam, Germany

**Keywords:** Pain occurrence, Young athletes, Injury, Training volume

## Abstract

**Background:**

Recently, the incidence rate of back pain (BP) in adolescents has been reported at 21%. However, the development of BP in adolescent athletes is unclear. Hence, the purpose of this study was to examine the incidence of BP in young elite athletes in relation to gender and type of sport practiced.

**Methods:**

Subjective BP was assessed in 321 elite adolescent athletes (m/f 57%/43%; 13.2 ± 1.4 years; 163.4 ± 11.4 cm; 52.6 ± 12.6 kg; 5.0 ± 2.6 training yrs; 7.6 ± 5.3 training h/week). Initially, all athletes were free of pain. The main outcome criterion was the incidence of back pain [%] analyzed in terms of pain development from the first measurement day (M1) to the second measurement day (M2) after 2.0 ± 1.0 year. Participants were classified into athletes who developed back pain (BPD) and athletes who did not develop back pain (nBPD). BP (acute or within the last 7 days) was assessed with a 5-step face scale (face 1–2 = no pain; face 3–5 = pain). BPD included all athletes who reported faces 1 and 2 at M1 and faces 3 to 5 at M2. nBPD were all athletes who reported face 1 or 2 at both M1 and M2. Data was analyzed descriptively. Additionally, a Chi^2^ test was used to analyze gender- and sport-specific differences (*p* = 0.05).

**Results:**

Thirty-two athletes were categorized as BPD (10%). The gender difference was 5% (m/f: 12%/7%) but did not show statistical significance (*p* = 0.15). The incidence of BP ranged between 6 and 15% for the different sport categories. Game sports (15%) showed the highest, and explosive strength sports (6%) the lowest incidence. Anthropometrics or training characteristics did not significantly influence BPD (*p* = 0.14 gender to *p* = 0.90 sports; r^2^ = 0.0825).

**Conclusions:**

BP incidence was lower in adolescent athletes compared to young non-athletes and even to the general adult population. Consequently, it can be concluded that high-performance sports do not lead to an additional increase in back pain incidence during early adolescence. Nevertheless, back pain prevention programs should be implemented into daily training routines for sport categories identified as showing high incidence rates.

## Background

Recently, the rate of prevalence of back pain (BP) in young elite athletes has been reported at 5–66%, depending on the cohort and time period analyzed [1–4]. Therefore, point (8% [3]), 1-year (57% [4]) and lifetime prevalence (66% [4]) rates show that back pain is already relevant in young elite athletes. Compared to the general population, the prevalence of back pain in adolescent high-performance sports is assumed to be equal or lower [3–6]. In a recent meta-analysis, Calvo-Munoz [5] calculated a mean point prevalence of 12% (range: 3-35%; 10 studies; *N* > 40,000) in children and adolescents. Balagué et al. showed a mean point prevalence of 13% in adolescent schoolchildren aged 10 to 16 years (*N* > 600) [7]. Additionally, Ellert et al. reported a gender-specific difference of 10% in back pain prevalence in children and adolescents, with girls showing higher rates than boys [6]. In contrast, Müller et al. found no relevant gender differences in back pain prevalence in adolescent athletes [3].

Regarding sports, prevalence rates vary depending on the study [1, 3, 4]. Schmidt et al. reported varying prevalence rates in different types of sports, with significant differences in lifetime prevalence rates between biathlons and volleyball in adolescent athletes [4]. Hence, prevalence is reported at between 30 and 80% in gymnasts and up to 27% in football [1, 8]. In contrast, we presented prevalence rates ranging from 3% (soccer) to 14% (canoeing) [3]. Prevalence rates in weight lifting, judo, wrestling, rowing, and shooting were ≥10%, while boxing, soccer, handball, cycling and horse riding were ≤6%. These results indicated that game sports (soccer, handball, volleyball) carried a lower risk of back pain (point prevalence) compared to all other sports disciplines. In contrast, combat sport athletes showed the greatest prevalence.

Despite the prevalence data, no study is known to have investigated the incidence of back pain in young elite athletes or even in adult athletes in a longitudinal setting. Analyzing the general population, Kopec et al. [9] reported an incidence rate of back pain in adults of 9% without gender differences over a 2-year period. In contrast, Cassidy et al. showed an incidence rate of 18% in a comparable population over a 1-year period [10]. In adolescents, Burton et al. identified a 1-year incidence rate of 21% in 15-year-old non-athletes [11].

The purpose of the study was to examine the development (incidence) of back pain in adolescent athletes with respect to anthropometrics and sport characteristics over a mean period of 2 years of systematic training.

## Methods

### Participants

In total 343 athletes from the elite schools of sports throughout the local federal state were recruited for the study. Due to back pain at measurement day M1, 22 athletes were excluded from the incidence analysis. Finally, 321 (183 males/138 females) participants with a mean age of 13.1 ± 1.4 years, all free of back pain at M1, were included in the data analysis. Anthropometrics and training data (for both measurement days) are detailed in Table 1. The athletes were recruited from 19 different sports (bob (*n* = 1), boxing (*n* = 11), soccer (*n* = 41), artistic gymnastics (*n* = 4), weight lifting (*n* = 9), handball (*n* = 28), judo (*n* = 15), canoeing (*n* = 17), karate (*n* = 1), athletics track & field (*n* = 32), modern pentathlon (*n* = 7), cycling (*n* = 16), horse riding (*n* = 45), wrestling (*n* = 35), rowing (*n* = 24), swimming (*n* = 17), shooting (*n* = 5), triathlon (*n* = 2), volleyball (*n* = 11)).

**Table 1:** Anthropometric and training data for all athletes on both measurement days (M1/M2)

Measurement day	n	Gender (m/f) [%]	Age [yrs]	Height [cm]	Body weight [kg]	Training years [yrs]	Training hours per week [h/week]
M1	321	57/43	13.2 ± 1.4	163.4 ± 11.4	52.6 ± 12.6	5.0 ± 2.6	7.6 ± 5.3
M2	321	57/43	15.2 ± 1.1	171.4 ± 9.8	62.0 ± 11.7	6.4 ± 2.8	13.4 ± 5.8

Stat. signif. differences for age, height, weight, training yrs and training hours (*p* = 0.001) between M1/M2

### Procedure

**Fig. 1:**
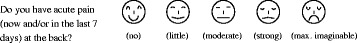
Standardized questionnaire with a 5-stepped graded face scale to assess back pain in young athletes

A prospective study design with two measurement days was used to evaluate the rate of back pain incidence in adolescent athletes. In general, measurement day 1 (M1) was conducted before entry to an elite sports school, and measurement day 2 (M2) after 2.0 ± 1.0 years of being an athlete at this type of school. As part of the annual pre-participation examination of upcoming and current athletes in the elite sports schools, subjective back pain was assessed twice (M1 and M2) in all athletes with a standardized questionnaire [3]. Back pain was defined as acute pain present at the time of answering the questionnaire and/or during the 7 days prior to the examination (Fig. 1) [12]. Additionally, anthropometrics (age, gender, height, weight) as well as sport type and training characteristics were assessed. All participants and their legal guardians were informed of the study and the questionnaire in a personal conversation with the principle investigator and through written study information. Subsequently, the children and their legal guardians provided written informed consent. The University of Potsdam’s Ethical Committee approved all procedures conducted during the study.

### Outcome measures and data analysis

Subjective back pain was assessed with a standardized questionnaire consisting of a 5-step face scale [6, 13, 14]: face 1 = no pain, face 2 = little pain, face 3 = moderate pain, face 4 = strong pain, face 5 = maximum imaginable pain. This type of questionnaire has been described as valid for the use of pain assessment in children and adolescents [6, 13, 14]. According to Merrati et al. [14] and Müller et al. [3], faces 1 and 2 are interpreted as no pain and faces 3 to 5 as pain.

The main outcome measure was the incidence rate of back pain [%] as determined by the development of back pain from M1 to M2. Participants were classified into athletes who developed back pain (BPD) and athletes who did not develop back pain (nBPD). BPD included all athletes who reported faces 1 and 2 at M1 and faces 3 to 5 at M2. nBPD were all athletes who reported face 1 or 2 at both M1 and M2 [14]. Anthropometric data for both groups are detailed in Table 2.

**Table 2:** Anthropometric and training data for BPD and nBPD on both measurement days (M1/M2)

Group	Measurement day	n	Gender (m/f) [%]	Age [yrs]	Height [cm]	Body weight [kg]	Training years [yrs]	Training hours per week [h/week]
nBPD	M1	289	56/44	13.1 ± 1.4	163.2 ± 11.3	52.2 ± 12.5	5.0 ± 2.6	7.6 ± 5.3
	M2	289	56/44	15.1 5 ± 1.1	171.1 ± 10.0	61.5 ± 11.6	6.4 ± 2.8	13.4 ± 5.8
BPD	M1	32	69/31	13.3 ± 1.2	165.9 ± 11.4	55.8 ± 12.9	5.0 ± 2.6	7.0 ± 4.8
	M2	32	69/31	15.3 ± 0.8	174.0 ± 8.2	66.2 ± 11.7	6.6 ± 2.4	14.1 ± 5.9

In addition, 4 different sport categories, combat sports (A: *n* = 62; boxing, karate, judo, wrestling), game sports (B: *n* = 80; soccer, handball, volleyball), explosive strength sports (C: *n* = 53; bob, artistic gymnastics, weight lifting, athletics track & field, modern pentathlon) and endurance sports with some strength components (D: *n* = 126; canoeing, cycling, horse riding, rowing, swimming, shooting, triathlon) were formed based on the main load type of each sport.

Back pain incidence was analyzed descriptively. Additionally, a Chi^2^ test was used to analyze gender- and sport-specific differences, followed by a logistic regression analysis including anthropometrics (gender, age, height, weight) and sports/training variables (sport discipline, sport categories, training volume, years of training) (α = 0.05).

## Results

**Fig. 2:**
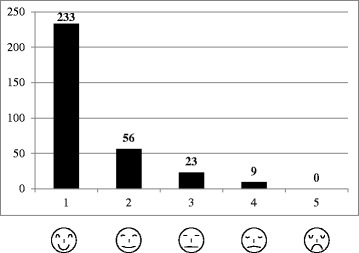
Number (frequency: [n]) of athletes per category of the face scale at M2

At M1, 86% of the athletes reported no pain (face 1) and 14% only little pain (face 2). All these athletes were categorized as free of pain at the time of inclusion into the study. At M2, 73% of the athletes reported no pain (face 1), 17% little (face 2), 7% moderate, 3% strong (face 4) and 0% maximum pain (face 5) (Fig. 2).

Categorized into the two groups (BPD/nBPD), 32 athletes reported pain (faces 3 to 5) at M2 and were therefore assigned to the BPD group, representing an overall back pain incidence rate of 10%.

### Back pain incidence and gender

Ten (BPD) out of 138 female athletes reported back pain (7%) at M2. In contrast, 22 (BPD) out of 183 male athletes reported back pain (12%) at M2. The gender difference was 5%, which was not statistically significant (*p* = 0.15).

### Back pain incidence and sport types

**Fig. 3:**
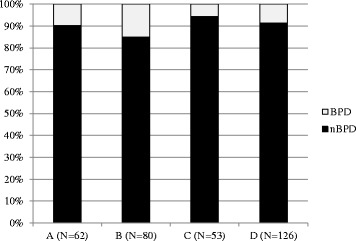
Rate of “Back Pain Developers” (BPD) and “No Back Pain Developers” (nBPD) [%] in the 4 sport categories (A: combat; B: game; C: explosive strength; D: endurance)

The analysis of the different sport categories (A, B, C, D) revealed that game sports showed the highest (15%) and explosive strength sports the lowest (6%) incidence of back pain (Fig. 3). The range of incidence rates, regarding single sport types, were between 0% (e.g. artistic gymnastic) to 27% (volleyball). Differences between the sport categories were not statistically significant (*p* > 0.05). In addition, the absolute numbers of athletes developing pain (BPD) or not (nBPD) are detailed in Table 3 for each type of sport.

**Table 3:** Number (frequency: [n]) of athletes categorized as “No Back Pain Developers” (nBPD) and “Back Pain Developers” (BPD) in each sport

Category	Sport	nBPD (n)	BPD (n)	Incidence [%]
A: combat sports	Boxing (*n* = 11)	9	2	18
	Karate (*n* = 1)	1	0	0
	Judo (*n* = 15)	13	2	13
	Wrestling (*n* = 35)	33	2	6
B: game sports	Soccer (*n* = 41)	35	6	15
	Handball (*n* = 28)	25	3	11
	Volleyball (*n* = 11)	8	3	27
C: explosive strength sports	Bob (*n* = 1)	1	0	0
	Artistic gymnastic (*n* = 4)	4	0	0
	Weight lifting (*n* = 9)	8	1	11
	Athletics track & field (*n* = 32)	30	2	6
	Modern pentathlon (*n* = 7)	7	0	0
D: endurance sports with strength component	Canoeing (*n* = 17)	15	2	12
	Cycling (*n* = 16)	15	1	6
	Horse riding (*n* = 45)	42	3	7
	Rowing (*n* = 24)	20	4	17
	Swimming (*n* = 17)	16	1	6
	Shooting (*n* = 5)	5	0	0
	Triathlon (*n* = 2)	2	0	0

Finally, the logistic regression analysis could not identify a statistically significant influence of any anthropometric or sport variable (*p* > 0.05; r^2^ = 0.0825).

## Discussion

The purpose of the study was to analyze the incidence of back pain in young elite athletes from different sports. The main results showed an incidence of 10% for the assessed systematic training interval, without statistically significant anthropometric, gender or sport-specific differences.

Using the assessment and categorization system of the face scale (nBPD (faces 1–2)/BPD (faces 3–5)) our 10% back pain incidence rate in young elite athletes was lower compared to that of young non-athletes of the same age (1-year incidence: 21% [11]). Kopec et al. [9] reported a 2-year incidence of back pain of 9% in an adult population, which is in line with the results of this study. Even taking into account a possible underestimation of our incidence rate due to the 7-day back pain prevalence scale used here, the comparison to young non-athletes and even to the general adult population confirms that high-performance sports do not lead to a relevant increase of back pain incidence in adolescence [9, 11, 15]. Nevertheless, the development of back pain (incidence) in the growing athletes has to be discussed against the background of an increased number of systematic training hours per week compared to their starting point at an elite sports school.

Regarding gender, Schmidt et al. [4] and Müller et al. [3] reported no gender differences in back pain prevalence in adolescent athletes. In comparison, Ellert et al. [6] reported gender differences between 5% (at age 11) and 10% (at age 16) in adolescent non-athletes. So far, no incidence rates of back pain are known in the literature for adolescent athletes. In line with the results from Kopec et al. [9] for adults, the incidence of back pain in the cohort presented here did not show statistically significant gender differences among young athletes (≤5%). Nevertheless, a 5% higher incidence rate in males might be discussed as clinically relevant for sports medicine staff and team physicians to address.

Detailed analysis of the sport types indicated that game sport athletes (soccer, handball, volleyball) might have a higher risk of developing back pain (incidence) compared to the other categories analyzed here. In contrast, athletes from explosive strength sports showed the lowest incidence, but with the small sample size for BPD, no statistically significant influence could be proven. In the literature, it appears that sports with a high amount of translation, extension and rotation (e.g. volleyball, soccer, handball, judo, wrestling, weight lifting) along with inadequate compensation of high loading will increase the risk of back pain [16–20]. This is partly in line with the results of the study presented here, where athletes of these disciplines (soccer, volleyball, handball) showed a higher incidence of back pain. As a consequence, special intervention programs aimed at preventing back pain should be implemented in the training routine.

Beyond this, pain intensity and thus the categorization system of BPD and nBPD groups has to be discussed as a relevant factor in back pain incidence in adolescent high-performance sports. Taking all pain athletes – independent of their grade of pain intensity (starting at face 2 on the scale) - into account, incidence rates increased by a factor of almost 2 (21%). This resulted in a similar incidence rate compared to that of adolescent non-athletes of the same age [11]. The face scale used here is described in the recent literature and is recommended as appropriate for pain assessment in adolescents [14].

Some methodological considerations have to be discussed. Point prevalence was used to answer the research question, disregarding such episodes of back pain as those occurring during training, for example. Therefore, the data might have underestimated the total incidence rate in the athletes. In the current literature, different definitions of point prevalence are used, with different time frames analyzed (24 h to 7d [4, 5, 12]. In this study, we defined point prevalence as pain at the time point of answering the pain questionnaire including the previous 7 days [12]. In addition, while the prevalence of back pain is generally often assessed with closed Yes-or-No questions [4, 5, 12], pain assessment in this study was conducted with a valid and reliable face scale to assess pain in children and adolescents [13, 14]. Admittedly, the different time frame between the first (M1) and second (M2) assessments in the individuals (high SD) was a limitation, but on average no difference between the BPD and nBPD groups was found. Also, the location of back pain, e.g., lower back, upper back, etc., was not specified in this questionnaire. The location of the pain might vary among individuals from different sport disciplines, which would influence the eligibility of certain intervention strategies. Therefore, the interpretation of the results needs to take these differences into account.

## Conclusion

Back pain development in young elite athletes has to be considered relevant, in spite of a lower incidence rate compared to adolescent non-athletes. It could be determined that high-performance sports did not lead to statistically significant increase of back pain incidence in adolescence. Nevertheless, back pain often leads to disability and, therefore, the need for a back pain prevention program in young athletes. Especially in game sports, prevention programs focusing on trunk stability to resist high training loads should be developed, validated and implemented into the daily training routine.

## Abbreviations

A: Combat sports
B: Game sports
BP: Back pain
BPD: Back pain developers
C: Explosive strength sports
D: Endurance sports with strength component
nBPD: No back pain developers
